# Loneliness in sports active and non-active school-age children: Can sport protect children against loneliness?

**DOI:** 10.3389/fpsyt.2022.1063714

**Published:** 2023-01-11

**Authors:** Tatjana Tubić, Toni Modrić, Damir Sekulić, Antonino Bianco, Izet Radjo, Patrik Drid

**Affiliations:** ^1^Faculty of Sport and Physical Education, University of Novi Sad, Novi Sad, Serbia; ^2^Faculty of Kinesiology, University of Split, Split, Croatia; ^3^Sport and Exercise Sciences Research Unit, University of Palermo, Palermo, Italy; ^4^Faculty of Sport and Physical Education, University of Sarajevo, Sarajevo, Bosnia and Herzegovina

**Keywords:** loneliness, sports participation, school-age children, prevention, mental health

## Abstract

**Introduction:**

This study aims to examine can sport protect children against loneliness and, if yes, whether this relationship depends on gender and/or sports-related variables.

**Methods:**

The sample includes 762 10-year-old children (414 boys). The total score of The Children's Loneliness Scale is a dependent variable in the study, while sports participation (sports active—individual or team sports, or non-active) and level/duration of sports participation are independent variables.

**Results:**

Obtained results indicate that the degree of loneliness differs depending on gender and whether the children are in sports activities or not. Unlike the level of sports participation, the duration of sports participation is relevant to the loneliness degree in both boys and girls.

**Discussion:**

This research results could represent the right direction for educators and/or parents in their endeavor to preserve and develop school-age children's mental health.

## 1. Introduction

Loneliness is one of the substantial problems of today's man, more present than ever before; moreover, loneliness may be considered a growing health epidemic (1, 2). In this study, loneliness is placed in the context of sports psychology in order to examine how loneliness relates to sports participation in school-age children and can sport really protect children against loneliness.

Previous findings concerning loneliness, in general, represent the necessary preconditions for their implementation in a particular area. Loneliness is a subjective experience or, more specifically, subjective state of negative feeling about having a lower level of social contact than desired (3); a person may be socially isolated but not lonely, or socially connected but feel lonely. Thus, loneliness is synonymous with perceived social isolation, not with objective social isolation (4). Although loneliness arises when our social needs are unmet by both the quantity and quality of our current social relationships, loneliness is more closely related to qualitative than quantitative aspects of social relationships (3, 5).

Loneliness can be harmful to physical health; loneliness may cause an increase in blood pressure, cholesterol, and risk of cardiovascular disease, which can lead to earlier death (6). In addition, it was found that lonely individuals were more likely to be smokers and/or overweight (7). It should be emphasized that loneliness in children has “persistent and cumulative detrimental effects on adult health” (p. 805), as proved in a (20-year) longitudinal study (8). Persistent loneliness is a powerful trigger for mental health problems, too. Research suggests that loneliness increases depressive symptoms, fear of negative evaluation, anxiety, and stress while diminishing optimism and self-esteem (4). Undoubtedly, this is a bi-directional relationship: anxiety and/or depression make social interaction difficult as well as unsatisfactory interaction leads to anxiety and depression.

Contrary to common stereotypes that loneliness is restricted to old age, studies revealed that these difficulties in connecting with other people could be experienced at any age (9). One-in-five children aged 7–12 report feelings of loneliness sometimes or often (10), while four-out-of-five adolescents say the same (11). For example, if the teenager has two friends, she may feel lonely, but if her grandmother has two friends, she will be fulfilled in terms of connectedness, which testifies to age differences in loneliness. Loneliness is present from the earliest days, growing during childhood and adolescence (12).

A recent meta-analysis indicates that there are no gender differences in loneliness across childhood and adolescence. Moreover, regarding loneliness, males and females are more alike than they are different across the whole lifespan (13, 14).

Studies have found that antecedents of loneliness and their consequences have a high degree of overlap (15); lack of physical activity seems to be one of these certain behaviors which can be both influencing factors on loneliness and a repercussion of loneliness (16). Namely, physical activity and/or sports participation imply and encourage interaction; during physical activity, individuals share similar experiences and interests, opportunities are created for establishing stronger ties, etc., which satisfies their social needs and protects them from loneliness. At the same time, the changes that occur in self-perception during physical activity, especially in social competence (17, 18), as well as the good feeling effect associated with the increases in serotonin, monoamine and neurotrophin production (19, 20) should not be ignored. If, however, an individual does not engage in physical activity or sport, he or she denies himself/herself most of the listed opportunities for establishing contacts and inclusion in the group, which can lead to loneliness (18, 21). Physical activity or inactivity, therefore, can be the antecedent of loneliness.

On the other hand, a lonely individual, over time, becomes hypersensitive to stimuli coming from the environment, perceiving them as potentially threatening (5). This reflects the interpretation of everything that comes from that social environment, so he/she focuses more on negative social interactions than on positive ones and remembers mostly negative feedback. Objectively negative social experiences are only perceived as a confirmation of their negative expectations, which further results in an individual's distancing from others while attributing the cause of that distance to others. That way, loneliness leads to avoiding even contact through sports activity and thus, negatively affects their physical activity (22).

A systematic review of loneliness and physical activity in a sample of 36 studies confirms that physical activity can contribute to a decrease in loneliness but also that loneliness itself may reduce the probability of being physically active (16).

What simply arises as the question is, how to get out of this vicious cycle? If physical activity and/or sport can protect against loneliness, is it enough just to be physically active, or some sports-related variables are also significant? Along with whether the preventive power of sports participation on loneliness depends on gender?

Previous research, less focused on mental health outcomes of participation in physical activity and more on physical health benefits (16, 23), offers inconsistent answers to these questions. Among the small number of studies that deal with the relations between sport and loneliness (and not physical activity and loneliness), even fewer research examines the relations between loneliness and specific variables that more closely determine sports activity. What is more, they were conducted on different age samples, as well as by applying different measures of loneliness, which makes comparing the obtained results difficult.

What also represents limitations in previous findings concerns the fact that a very small number of studies on the relationship between loneliness and sport, about 10%, are conducted in children samples (16). If it is known that loneliness in childhood has been linked with lower school liking, school dropout, social anxiety, and depression (18, 24), as well as less physical activity later in adulthood (8), which is, again, associated with numerous diseases (25), then the school-age sample deserves full attention.

However, from these researches, it can be stated that participating in team sports reduces loneliness to a greater extent than participating in individual sports (23). Additionally, the effects of sports participation on the reduction of loneliness are more pronounced in females than in males (26, 27).

With all of the above in mind, this study starts from the following:

- that the sample will consist of school-age children of both gender;
- the focus will be “purely on the emotional experience of loneliness without including hypothesized causes” [(28), p. 6];
- Thus understood, loneliness will be associated with participation in sport, bearing in mind that sport is a narrower term than physical activity and implies one type of leisure-time physical activity that is organized, usually competitive, and practiced in a team or individually (23).

Hence, this paper aims to analyze the relationship between loneliness and sports participation among school-age children. More precisely, it examines if children differ in loneliness depending on gender and/or whether they participate in sport (individual or team) or not and if experience in sport and/or level of sport participation contributes to obtained results in their loneliness. Based on previous findings about loneliness in children, we hypothesize that sports can protect children against loneliness and that the understanding of the relationship between loneliness and sports participation can be contributed by examining gender and/or sports-related variables in our particular subsamples.

## 2. Materials and methods

### 2.1. Participants

The sample consists of 762 fifth-grade students from eight urban and rural primary schools in the Autonomous Province of Vojvodina, Serbia. The structure of the sample by gender, sports (non-) activity, as well as by type of sport (individual or team) is given in Table 1.

**Table 1:** Sample and subsamples depending on gender and sports (non-) activity.

		**Sports participation**				
		**Sports active**				
		**Individual** **sport**	**Team** **sport**	**∑Sports** **active**	**Non-active**	**∑**
Boys	*N*	104	225	329	85	414
	%	31.6	68.4	79.47	20.53	54.33
Girls	*N*	109	122	231	117	348
	%	47.2	52.8	66.38	33.62	45.67
Total sample	*N*	213	347	560	202	762
	%	38.00	62.00	73.49	26.51	100

Within the subsample of boys who participate in sport, 92 participate at the international level, 57 at the national level, 52 at the provincial level, 53 at the district level, and 75 at the city level.

In the same subsample, 55 participate in sport for up to a year, 75 between 1 and 3 years, and 199 for more than 3 years. As for girls who participate in sports, 48 participate at the international level, 25 at the national level, 32 at the provincial level, 39 at the district level, and 87 at the city level. In the same female subsample, 60 participate in sport for up to a year, 72 between 1 and 3 years, and 99 for more than 3 years.

### 2.2. Research procedure

Regarding the research procedure, the study was conducted just before the COVID-19 pandemic (fall, 2019); first of all, the research assistants introduced the homeroom teachers in the schools with the main objective of the research and in cooperation with them agreed on the testing dates in the school itself, most often during homeroom class. Then, at the parent-teacher meeting, those teachers informed the parents about the planned testing and got their permission. Before testing, the researchers—briefly and age-appropriately—introduced the children to the main research objective; the importance of an honest answer, as well as the clarification of any doubts on the spot, was emphasized. Participation was voluntary; all examinees were informed that no personalized data would be used in the analyses and that no personalized results would be obtained, since all data were assembled on group level. Additionally, a maximum of 30 children was tested together in the classroom. Administration of the total protocol took ~20 mins. All schools were tested within 4 weeks.

### 2.3. Instruments

The Children's Loneliness Scale (CLS), originally referred to as the Loneliness and Social Dissatisfaction Questionnaire, is the first scale developed to assess feelings of loneliness in childhood, designed by Asher and Wheeler (29). Both it's the original version which is intended to measure loneliness in 3rd through 6th graders' (1), and their adapted versions to measure loneliness from preschool to Grade 2 (30) as well in the school context (29), the measure has established itself as *the gold standard* in the measurement of childhood loneliness (31).

In this study is used the original version of the Children's Loneliness Scale, considering that loneliness is conceptualized as a global construct which means that feeling lonely is related to the entirety of an individual's existing interpersonal relationships; the scale focused on school-related aspects of loneliness would be more context-related (16).

This 24-item scale comprises 16 primary items designed to tap into children's feelings of loneliness and 8 filler items on children's hobbies and preferred activities and school subjects. All items were responded to on a five-point Likert-type scale; a loneliness score is calculated based on 16 primary items and could range between 16 and 80. Higher scores reflect higher degrees of loneliness. Internal consistency for CLS was estimated at 0.90 (1).

In the present study, the tool has been translated into Serbian and the internal consistency was α = 0.903; test-retest reliability calculated with an interval of 100 days is *r* = 0.577, *p* < 0.00. Accordingly, these psychometric properties showed that the CLS has high internal reliability and provides stability in measuring the loneliness in Serbian 10-year children.

In addition to the total score on the Children's Loneliness Scale, which is a dependent variable in this study, independent variables are gender, sports participation (sports active or non-active), type of sports participation (individual or team sport), level of sports participation (city, district, provincial, national or international) and sports experience expressed in the number of years of sports participation (less than a year, between 1 and 3 years or more than 3 years).

### 2.4. Statistical analyses

Besides basic methods of descriptive statistics which were used for examining loneliness in school-age children (Mean, Standard Deviation, *t* test for independent samples), relations between their loneliness and sports participation was analyzed using One-way ANOVA (when having a single independent variable which is categorical, consisting of two or more levels) and Univariate Analysis Variance in General Linear Model (when having more than one independent variable) and, a *post-hoc* test as well, to find where the significance between groups exist; in Univariate Analysis Variance procedure, gender and sports participation (sports active and non-active; individual, team sport and non-active) are treated as Fixed Factors, while experience in sport and level of sports participation are seen as Covariates.

## 3. Results

Results of Univariate Analysis of Variance in the total sample indicate that the degree of loneliness differs depending on *gender* and whether the children are *sports active or not* (*F* = 7.421; *p* = 0.007). By insight into relationships within the examined system of variables, it was found that primary school boys and girls do not differ in loneliness. However, there are statistically significant differences between children who participate in sports and those who do not, where sports inactive children are found to be more lonely (*t* = −2.211; *p* = 0.027). In addition, it was found that boys who do not participate in sports are significantly more lonely compared to girls who do not participate in sports (*t* = 2.67; *p* = 0.008). At the same time, boys who do participate in sports do not differ significantly from girls who are in sports activities in terms of loneliness.

As shown in Table 2, attention is also paid to determining whether and to what extent the type of sport that children participate in can be related to their loneliness degree. When using One-way ANOVA for comparing children who participate in individual or team sports with children who are not sports active, statistically significant differences in loneliness are found (*F* = 3,819; *p* = 0.022). Children who participate in team sports are found to be statistically significantly less lonely than children who are not sports active (*t* = −2.758; *p* = 0.006). Differences in loneliness between children who participate in an individual sport and those who are not sports active, as well as between children who participate in an individual sport and in a team sport, are not statistically significant.

**Table 2:** Loneliness depending on gender and sports (non-) activity.

		**Sports participation**				
		**Sports active**				
		**Individual** **sport**	**Team** **sport**	**∑Sports** **active**	**Non-active**	**∑**
Loneliness in boys	M	26.30	24.29	24.92	29.42	25.85
	SD	11.07	8.73	9.56	12.49	10.38
Loneliness in girls	M	25.72	24.93	25.30	25.15	25.25
	SD	11.12	9.60	10.33	10.18	10.26
Loneliness in total sample	M	26.00	24.52	25.08	26.95	25.58
	SD	11.073	9.04	9.88	11.38	10.32

F = 3.566; p = 0.029.

Since the Univariate Analysis of Variance indicates that there are statistically significant differences in loneliness depending on gender and whether children participate in an individual sport, team sport, or they are not sports active at all (*F* = 3.566; *p* = 0.029), in the continuation of the chapter the results for children who do not participate in sport or participate in individual or team sports at different levels or for different durations will be presented separately for the boys' and the girls' subsamples.

### 3.1. Loneliness in sports-active and non-active boys

Considering that boys who do not participate in sport is as a subsample that is statistically significantly more lonely than boys who participate in sport (*t* = −3.614; *p* = 0.000), the question is, first of all, whether there are differences between boys who participate in an individual sport, in team sport and those boys who do not participate in sport.

The results of the One-way ANOVA confirm that the differences in the degree of loneliness among boys who participate in an individual sport, team sports and those who do not participate in any sport are statistically significant (*F* = 7,929; *p* = 0.000). *Post-hoc* analysis indicates that, as in the case of the total sample, these differences in loneliness are statistically significant between boys involved in team sport and those not involved in sport (*t* = −4.071; *p* = 0.000), while differences between boys who participate in an individual sport and those who are sports non-active are not significant, nor are those obtained between boys engaged in individual and team sports.

Further, it should be investigated whether the level and/or duration of sports participation can contribute to feeling less lonely in subsamples of boys who participate in team or individual sports. Univariate Analysis of Variance suggests that the differences in loneliness between boys who do not participate in sport, the ones who participate in an individual sport, and the ones who participate in a team sport *at different sports levels* and/or *duration* are statistically significant (*F* = 8.903; *p* = 0.000).

More specifically, the One-way ANOVA shows that there are no statistically significant differences in loneliness between boys who participate *in a team sport at different levels* (from city to international). *Post-hoc* analysis confirms this when comparing particular pairs of examined subsamples; there is no trend that would indicate more or less loneliness depending on the level of competition of boys engaged in a team sport. However, when it comes to the relation between loneliness and duration of team sport participation in boys (Table 3), it is very important how long they participate in these sports, as evidenced by statistically significant differences obtained using the One-way ANOVA (*F* = 3.706, *p* = 0.026).

**Table 3:** Loneliness in boys depending on their sports experience.

		**Sports experience**		
		**Less then** **a year**	**Between 1 and 3 years**	**More than 3 years**
Loneliness in individual sport	M	28.92	26.05	25.31
	SD	12.44	12.18	10.02
Loneliness in team sport	M	26.26	26.36	23.09
	SD	9.27	10.91	7.45
Loneliness in sport active boys	M	27.42	26.27	23.73
	SD	10.74	11.21	8.32

F = 3.706; p = 0.026.

Namely, *post-hoc* analysis reveals that boys who participate in a team sport for *more than* 3 *years* are less lonely compared to boys who participate in a team sport *for 1–3 years* or *less than a year* and that these differences are statistically significant (*t* = 2.384, *p* = 0.018 and *t* = 2.051, *p* = 0.042, respectively). Boys who have been involved in a team sport *for less than a year* and *from 1–3 years* form a relatively homogeneous group in terms of loneliness. There are no significant differences in loneliness between them.

As for boys who participate in individual sports, the One-way ANOVA indicate that neither their sports level nor the duration of engagement in that sport is not significant for their degree of loneliness; no statistically significant differences were obtained in the case of any single pair of subsamples.

### 3.2. Loneliness in sports-active and non-active girls

In contrast with boys, Univariate Analysis of Variance on the overall system of examined variables indicates that differences in loneliness between girls who do not participate in sports or participate in individual or team sports at different levels and/or duration are not statistically significant.

Accordingly, the One-way ANOVA confirmed that girls do not differ from each other in loneliness degree depending on whether they participate in an individual sport or team sport or do not engage in sport et al. Regardless of *the level at which they participate in a team sport*, from city to international level, this does not significantly affect their loneliness. The same applies to sports experience: *the number of years in team sport* does not determine the extent to which girls will feel lonely.

Considering the loneliness of girls who participate *in an individual sport, the sports level* did not prove to be relevant, as well as *sports experience*, as shown by applying the One-way ANOVA test. By comparing particular pairs of subsamples respecting duration in the individual sport of girls, however, it was found that between those who participate in individual sport *for up to 1 year* (M = 29.31, SD = 11.39) and those who practice it *for more than 3 years* (M = 24.27, SD = 10.32) there are statistically significant differences in the degree of loneliness (*t* = 2.065; *p* = 0.042). In other words, individual sports can protect girls from loneliness if they practice them for at least 3 years.

## 4. Discussion

This study unambiguously reveals that practicing sports can protect children against loneliness. Whether that will be the case or not, and if yes, to what extent, depends on various factors.

First of all, the results suggest that *boys and girls do not differ in loneliness*. Obtained results are in contrast with Sexual selection theory (32) which links females with the development of internalizing problems, such as loneliness. Nevertheless, they are in line with the gender similarities hypothesis (13), which states that males and females are similar in most of the psychological variables (but not in all). Considering Hyde leaves the possibility of varying these variables at a different age, it should be noted again that our sample consists of 10-year children. So, in this age sample, gender differences in loneliness are too small to be important compared with a within-gender variation. This represents noteworthy information to parents and teachers: every child can be lonely, regardless of gender; gender itself does not increase the probability of feeling lonely.

Second, *sports-active children are less lonely in comparison with children who are not sports-active*. What is common to research that examines the relationship between loneliness and physical activity is that loneliness and physical activity correlate negatively, from which arises that physical activity reduces loneliness (21, 22, 33). The problem remains, however, in the methodological inconsistency of these research which makes them difficult for comparison, as well as in the deficit of research conducted in the children population. One few (34) used the same measure as in our study (1) in a children's sample but with several fitness tests as independent variables of physical activity. Findings obtained in that research correspond with our results to a large degree; lonely children were less physically active than those who were not lonely, and loneliness is perceived as a barrier to physical activity.

Among possible explanations for why lonely individuals are less physically active stands out, one which claims that they do not possess the social skills necessary for functioning in a sports group (34). Additionally, the Social control theory (35) explains that because lonely individuals are in contact with a low number of people, there is no incentive for health-related behavior, like sports activity. Also, loneliness changes our cognition, emotions, and behavior, and solonely individuals' impaired self-regulation reduces their physical activity (22).

Next, study results indicate that *active sports boys are far less lonely in comparison with boys who are not sports active, while girls do not differ depending on sports (non-) activity*.

Previous research suggests that females gain more social and emotional benefits from sports activity than males (36, 37). Females may be more sensitive to interpersonal interactions and more aware of the possibility of meeting their needs for social interaction during adolescence through sports participation (38). In a longitudinal study that applied the same measure as in our study [but revised, (29)] on the sample of children aged 12–14, it was also found that physical activity is associated with lower levels of loneliness among females, but not among males (27). On the other hand, our study results are in contrast with what was mentioned—sports participation has a greater positive influence on boys than on girls concerning loneliness. Moreover, our results provide evidence that boys who are not in sports activities are a vulnerable subsample; they are more likely to be lonely than any other examined subsample (sports-active boys, sports-active and non-active girls).

It might be that females, through sport, look for a way to fulfill their needs for connectiveness and/or affiliative motive (38). However, females can also fulfill these needs through other activity which is based on interaction, so sports context is not necessarily crucial for them, as our study confirmed. Unlike females, males, primarily through sport, seek satisfaction from their need for competition and dominance (26). It is possible that in other age samples, males would find other approaches for meeting those needs, for example, the business environment, which could cause a change in gender differences in a sports context. In mentioned gender similarities hypothesis, Hyde (13) emphasizes that the gender differences “can vary substantially in magnitude at different ages and depend on the context in which measurement occurs” (p. 581), so it can be said that for 10-year children, participation in sport shows significance in manifesting gender differences in loneliness.

Further, *children who participate in team sports are far less lonely in comparison with children who are not sports active, while between children who participate in an individual sport and sports non-active children, there are no differences in the degree of loneliness*. By analyzing the psychological and social benefits of sports participation in children and adolescents in 3,668 publications in English from 1990 to 2012, Eime et al. (23), found that the most common effects of sports are improving self-esteem and social interaction, as well as decreasing depressive symptoms. Also, in this systematic review, it is pointed out that team sport, specifically, is linked with increasing health-related variables when compared with individual sports, which is in line with our results. The authors explain these results by the social nature of team sport itself (more players, interaction, stimulation, similar interests, goals, and experiences, etc.) or, in other words, by the fact that the benefits of sports participation on mental health are in team sport more expressed. For example, cross-sectional studies in children samples show that team sport participation is positively associated with social acceptance and perceived social acceptance, with effects of positive experiences (in coaching, skill development, peer support) and with reducing body dissatisfaction (39). Although the relations between participation in team sports and loneliness were not the objective of previous research, mentioned results reveal a trend that could be reflected in loneliness, too. Namely, if sport contributes to social acceptance, that is connected with feeling good about own status in that sports group and with a positive mood in general, which altogether may lead to favorable social self-perception and reduce the probability of loneliness.

In the explanation of the obtained inverse relationship between team sports participation and loneliness, it is useful to notice another factor that is not so present in other activities. Team sport cherishes belongingness. It seems that belongingness is that secret spice that protects from loneliness in a team sport. Evidently, loneliness and belongingness have in common the individual perception of connectedness (40). Still, loneliness is an emotional consequence of the unmet need to belong. Through sport, an individual develops a sense of belonging (to the team, to the club, teammates, etc.) which reduces the probability of feeling lonely. It does not mean, however, that an individual will not be lonely if the intensity of this need exceeds what he/she perceives to get through interaction.

What is substantial, this study's results suggest that participation in team sport does not have equal importance in preventing and/or reducing loneliness in boys and girls; *team sport participation is associated with lower levels of loneliness in the boy's subsample but not in the girl's subsample*. These results support the explanations that have already been given when the loneliness of sports active boys and children who participate in team sports was discussed. Hence, boys' participation in team sports leads to greater benefits for them in terms of prevention or reduction of loneliness than if they participate in an individual sport or are not sports active at all, which is an important guideline for parents and teachers.

*The level of sports participation has not proved relevant in the context of examining children's loneliness; this applies to both the total sample and the separate subsamples of boys and girls*. In explaining these results, attention should be paid to the age of the examinees; although sports participation itself leads to changes in personality, as well as participation in team sports (for boys), the level of participation in chosen sport obviously does not make any difference at the age of ten (yet?).

It is clear that an individual's higher level of sports participation implies a specific lifestyle (higher number of obligations, training hours, commitment, priority planning, etc.) which reflects on personality and, accordingly, represents the possible protective or risk factor. The prospective influence of sports participation level on loneliness, therefore, should not be totally rejected but rather examined at an older age.

The pedagogical implications that follow from the above are very useful for the prevention and/or reduction of loneliness in children. It is not necessary to reach a high level of competition to feel the effects of sport on loneliness. It is enough to just participate in sport. It should be emphasized that some other factors that determine if a person will reach a higher level of competition are not considered. Nonetheless, the responsibility in terms of preventing loneliness lies solely with the one who decides whether to participate in sport or not. It is exactly the importance of the attributions that individuals create about themselves and their social world in order to understand their loneliness at the very core of the cognitive approach to loneliness, especially Wainer's Theory of Attribution (41).

In contrast to the level of sports participation*, sports experience is relevant for reducing loneliness in children, both boys, and girls*. Our study reveals that *children who participate in sports for more than 3 years have significantly lower scores in loneliness* when compared with those who participate for a shorter period. Whether exactly “more than 3 years” is the time threshold for the benefits of sports participation on loneliness or not remains to be seen in future research that would predict a larger number of different time intervals, as well as a larger number of examinees in each of them. In one of a few studies that link loneliness with the duration of physical activity (measured differently from ours), Randall and Bohnert (26) found that boys who were active up to 3 h per week were less lonely, while those who were active more than 7 h per week were more lonely. In this regard, it would be interesting to examine adolescents or adults in order to determine whether the regularities noticed in the 10-year-old sample apply to them as well and what duration of sports participation would be optimal for coping with loneliness.

Our results suggest that the duration of sports participation is relevant to the degree of loneliness in both boys and girls but strongly depends on the type of sport they practice. Namely, *boys who participate in a team sport for more than 3 years will be less lonely than all other examined subsamples*. On the other hand, *girls who participate in individual sports for more than 3 years will be less lonely* than those who practice it for up to a year.

The fact that loneliness refers more to the qualitative than to the quantitative aspects of social relations (15), in a sports context has definitely provided us with a new point of view. Thank that now we are able to complement the explanation of the results mentioned earlier that children, especially boys, are less lonely if they participate in sport.

Participation in a team sport by the very nature of that activity (more players, greater availability of potential contacts), therefore, provides *the quantity* of interaction, while *the quality* of interaction may rather be achieved through the length of team sport participation. During a longer period of sports participation, not only are relationships established between players but also these relationships are strengthened. As a consequence, the quality of interaction is noticed after some time of participating in sport, and the quality of interaction, exactly, is, in fact, a contra indicator of loneliness. In other words, team sport participation represents a healthy behavior in which the boys who do participate in it for a longer period will be protected from loneliness.

Unlike boys, participation in a team sport, regardless of its level or duration, has not proved relevant for the degree of loneliness in girls. From that, it can be concluded that for girls, the quantity of interaction in sports is not necessarily a trigger for establishing closer contacts. On the contrary, since girls control loneliness better in individual sports, that would mean they directly establish the quality of interaction which later reinforces and deepens through the duration of sports participation; this whole process leads to lower levels of loneliness. Of course, here we conclude about the quality of interaction only indirectly, through the loneliness degree and the specificity of the examined (sports) activity, but the acceptability of this explanation would be worth examining in future research.

Regarding the limitations, our research was conducted on 10-year-olds, so only the expansion of age categories would allow the generalization of the observed trends on the relationship between loneliness and sports participation. In addition, the examined age contributes to the fact that the level of sports participation should be understood conditionally and the results obtained in this regard. Therefore, it remains an open question of what kind of cognitive and emotional changes should be expected in lonely athletes over time and how these changes would be reflected in the level of sports participation. It should also be noted that the number and way of forming categories related to sports experience were reflected in the obtained results: by planning a larger number of categories, more precise data would be obtained on the relationship between loneliness and the duration of sports participation; these relations proved to be relevant for understanding the importance of sports participation in the mental health context, both at this age and in the categories formed in this way.

It should not be neglected that at the examined age, girls are more mature than boys in terms of intellectual, social, and emotional development. Maybe that is why girls act as a relatively homogeneous group when examining loneliness. Girls can achieve good feelings about themselves in various types of activities, while for boys at that age, the most important reinforcement comes from significant others, i.e., from the team. Girls, in addition, when engaged in individual sports for a longer period, adopt a model of behavior that requires them to find strength and motivation in themselves, protecting them from loneliness. Future research should focus on identifying sports-related factors that lead to different significance in protecting against loneliness for girls and boys. With all of the above, the study was conducted before the COVID-19 epidemic, but the lockdowns affected children's mental health and feelings of loneliness (42), so the results obtained are valuable especially after the COVID-19 epidemic.

## 5. Conclusion

This study examines relations between loneliness and children's sports participation with the intention to understand the contribution of sport in protecting and improving children's mental health. Although the problem of mental health is widespread, it is still unduly neglected as a research phenomenon among children. Likewise, loneliness in children does not get enough attention, especially in low-income and middle-income countries, but is the focus of this study. Therefore, these results could be helpful in planning interventional programs based on physical activity.

The essential finding is that sports participation in childhood can protect against loneliness. By virtue of sports activity, which provides children to meet their social needs and expectations, sports-active children are less lonely.

For mental health practitioners, educators, sports coaches, of course, parents, as well as everyone involved in professional and private interaction with children, the main value of this study is the fact that it could (and should) be used as a guideline for preventing and/or reducing loneliness in school-age children. Bearing in mind that team sport protects boys, while individual sport protects girls from loneliness, it is not the same which sport the lonely child will decide to take up. For example, now we know that a good choice for a lonely 10-year-old boy would be a team sport. We may even claim that the mentioned boy, who is lonely by his own or relevant professionals' estimate, should be advised to embrace team sport, regardless of the sports level (that he is able to achieve). Additionally, the finding teaches us that once the boy is directed toward team sport or a girl toward individual sport, the one has to be aware of the time needed (according to this study, at least 3 years) for the recommendation to give effects.

## Data availability statement

The raw data supporting the conclusions of this article will be made available by the authors, without undue reservation.

## Ethics statement

The studies involving human participants were reviewed and approved by University of Novi Sad, Faculty of Sport and Physical Education, Serbia (Ref. No. 46-06-02/2020-1). Written informed consent to participate in this study was provided by the participants' legal guardian/next of kin.

## Author contributions

Conceptualization: TT and PD. Writing—original draft preparation: TT. Writing—review and editing: TT, TM, DS, AB, IR, and PD. Visualization: TM. Supervision: DS. Funding acquisition: PD. All authors have read and agreed to the published version of the manuscript.
